# The Role of Stress, Social Norms and Attitudes in Help-Seeking for Nurses

**DOI:** 10.1155/jonm/8897678

**Published:** 2025-09-18

**Authors:** Niall Galbraith, David Boyda, Danielle McFeeters, Darren Chadwick, Wendy Nicholls, Debbie Stevens-Gill, Rachael Harris

**Affiliations:** ^1^School of Education & Psychology, Faculty of Education, Health and Wellbeing, University of Wolverhampton, MC Building, Wolverhampton WV1 1LY, UK; ^2^Early Intervention in Psychosis (West), Forward Thinking Birmingham, Birmingham Women's and Children's NHS Foundation Trust, 5th Floor, 1 Printing House Street, Birmingham B4 6DF, UK

**Keywords:** help-seeking, nurses, quantitative methods, stress, theory of planned behaviour

## Abstract

**Aims:** Explore the barriers to nurses' help-seeking for stress using the theory of planned behaviour.

**Background:** Perceived stigma can prevent health professionals from seeking psychological help, yet few studies have explored these barriers in the nursing profession.

**Methods:** After accounting for missing data, a total of 549 nurses were included in the final analysis. Measures included attitudes, subjective norms, perceived behavioural control, intention to seek help for stress and stress experienced in the previous 12 months. Analyses tested direct and indirect pathways between help-seeking beliefs and intentions, with stress as a moderator.

**Results:** Intention to seek help for stress was predicted by help-seeking attitudes and subjective norms. Perceived behavioural control did not predict help-seeking intentions. Attitudes and subjective norms were positively related to recent help-seeking behaviour and were mediated by current intentions to seek help. These mediated relationships were stronger in those who had experienced recent stress.

**Conclusions:** Workplace interventions to reduce the stigma of help-seeking for stress should target both personal attitudes in nurses but also cultural norms and values that prevail within healthcare organisations.

**Summary**

• Healthcare organisations can facilitate stress help-seeking among nurses by not only promoting positive attitudes but also improving organisational cultural norms towards seeking help.

## 1. Background

The importance of tackling stress and mental health in the health professions is increasingly recognised as a critical priority in the residual aftermath of the pandemic [1, 2]. Both individual-focused and organisation-focused solutions are effective in improving stress and well-being in health professionals [3–5]. However, for stress management in these groups to be successful, health professionals must first feel sufficiently confident and safe to disclose their stress and seek help when necessary [6]. Yet concerns are mounting over the mandatory mental health disclosures required for medical licensure, as these practices risk reinforcing the harmful perception that mental illness equates to professional incompetence [7]. The routine use of intrusive mental health questions in credentialing processes may, as a result, deter health professionals from seeking the support they need [2].

For example, research on help-seeking in other health professions such as doctors reveals a reluctance to disclose mental health difficulties to colleagues or employers, with treatment preferences motivated by career implications, stigma and confidentiality rather than quality of care [8–10]. In contrast, research on nurses' willingness to seek help for stress is sparse. This is particularly concerning given recent studies indicating elevated levels of stress and burnout among nurses across various specialties, including psychiatric, critical care and general medical settings [11, 12]. What does exist suggests that these individuals generally are reluctant to disclose or seek help to improve their mental health. Attitudes and social/cultural norms appear to play an important part in nurses' reluctance to seek help, with fear of career repercussions, perceived competence, negative social judgement, shame and stigma featuring strongly [13–16].

Evidence suggests that avoidance of help-seeking for mental health difficulties may be learned during training in health professionals [6, 17–19]. A reluctance to seek help amongst nurses is a worrying trend given the importance placed upon disclosure and help-seeking by recent high-profile initiatives such as the World Health Organization's (WHO) *Duty of Care* report and the CDC's and National Institute for Occupational Safety and Health's (NIOSH) *Impact Wellbeing* campaign [1, 2].

Thus, the available evidence suggests that not only are nurses subjected to significant levels of daily stress but that shared attitudes, and cultural norms may serve as impediments to seeking assistance. Given the significance of both individual and cultural variables in the management of occupational stress [20] and the relevance of social norms, attitudes and self-efficacy in the utilization of help [21], it is concerning that there are no comprehensive studies exploring the impact of personal and sociocultural factors on nurses' help-seeking behaviour in response to stress. To address this gap in the literature, the present study was designed, incorporating the theory of planned behaviour as its underlying theoretical framework.

The theory of planned behaviour [22] is a widely applied social cognitive theory modelling the attitude–behaviour relationship [23]. The theory of planned behaviour proposes that ‘intention' to carry out a behaviour is the proximal determinant and motivation behind volitional behaviour [22]. It also incorporates (i) ‘attitude' towards the behaviour, i.e., how positively or negatively the person evaluates that behaviour; (ii) the individual's perception of the social pressure they are under to perform the behaviour, termed ‘Subjective norms'; and (iii) ‘Perceived behavioural control'—the person's assessment of how easy or difficult they would find the behaviour. Behaviour is a function of intention and perceived behavioural control; intention is a function of attitudes, subjective norms and perceived behavioural control. Therefore, perceived behavioural control can influence behaviour through intention, but also directly, although this depends on how much actual control there is over the behaviour. Attitudes, subjective norms and perceived behavioural control are themselves dependent upon behavioural, normative and control beliefs.

The theory of planned behaviour has been utilised in numerous empirical studies to predict a variety of health behaviours, e.g., exercise and physical activity [24, 25], alcohol intake [26], healthy eating [27, 28] and sexual health [29]. The theory has been used to examine psychological help-seeking [30], but its application to nursing professionals' help-seeking remains relatively unexplored. The empirical literature has established that including additional moderating variables can enhance the explanatory power of the theory of planned behaviour [31]. This justification supports the inclusion of stress as a moderator in the present study. The transition from intention to actual help-seeking behaviour is unlikely to occur without the presence of stress, making it plausible that recent stress serves as a crucial moderator in the intention–behaviour relationship.

## 2. Aims

The present study aimed to utilise the theory of planned behaviour to examine help-seeking behaviours for stress among a sample of nurses in the United Kingdom. The model included attitudes towards seeking help for stress, subjective norms, perceived behavioural control over seeking help for stress and the intention to seek help for stress, as well as self-reported help-seeking behaviours. Given that the relationship between the intention to seek help and actual help-seeking behaviour may be contingent on perceived need, the frequency of stress experienced over the preceding 12 months was also included as a moderator in the relationship between intentions and behaviours.

**Figure 1:**
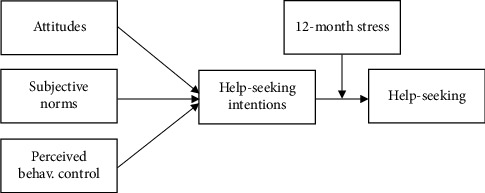
The conceptual model.

It was hypothesised that help-seeking beliefs (attitudes, subjective norms and perceived behavioural control) would exhibit a positive correlation with current intentions to seek help and with recent help-seeking behaviours (within the previous 12 months). Moreover, it was expected that current intentions to seek help would act as an intermediary in the relationship between help-seeking beliefs (attitudes, subjective norms and perceived behavioural control) and recent help-seeking behaviour. Finally, it was predicted that an interaction effect would be evident, such that the indirect relationships from help-seeking beliefs to recent help-seeking behaviour (via intentions) would be stronger when the frequency of recent stress experiences was higher (see Figure 1).

## 3. Methods

### 3.1. Participants

All study participants (*n* = 549) were members of the Nursing and Midwifery Council (NMC), a statutory regulatory body for nursing and midwifery in the United Kingdom, ensuring that all respondents were registered and fully qualified nurse professionals. An internal email invitation with a link to the online cross-sectional survey was distributed to all registered NMC members, detailing the study's purpose, voluntary participation and confidentiality.

### 3.2. Measures

#### 3.2.1. Demographic Questionnaire

The following demographic information was collected using open-ended questions: age, gender, level/grade (note: In the UK National Health Service, staff salaries are structured into bands ranging from 1 to 9) and ethnicity. A summary of the sample demographics is presented in Table 1.

#### 3.2.2. Theory of Planned Behaviour Questionnaire Development

The theory of planned behaviour questionnaire was constructed in line with the format instructions specified by Ajzen [32] and others [33] and represents all the variables described in the theory. The initial pool of items was elicited following consultation with health professionals and was subsequently tested on a sample of 146 student nurses from two different UK universities. Although student nurses are not entirely equivalent to qualified nurses, they were considered a suitable pilot sample given their placement experience in clinical settings. Moreover, previous research suggests that student and qualified nurses share similar attitudes towards work-related stress and help-seeking [34]. The items were reviewed for comprehensibility and following further discussions, two items on attitudes to help-seeking (*‘for me to seek help from a professional for stress would be: harmful-beneficial; pleasant-unpleasant'*) were removed. Furthermore, one item on subjective norms was re-worded to improve clarity (the term *‘approve of me'* replaced *‘support me'*).

#### 3.2.3. Attitudes Towards Help-Seeking

The question “Seeking *help from professionals for stress would be…*”, measured attitudes via seven bipolar scales each with evaluative opposing adjectives. These scales included a universal item (bad/good), an instrumental item (worthless/valuable) and five experiential items (shameful/admirable, unenjoyable/enjoyable, difficult/easy, foolish/wise, awful/nice). Using a nine-point Likert scale (*1 = strongly disagree* to *9 = strongly agree*), all items' scores were summed to derive a help-seeking attitude score and higher scores represented more positive attitudes to help-seeking (following item-score reversals where necessary). Both Cronbach's *α* (0.89), and McDonald's *ω* (0.88) indicated high internal consistency [35].

#### 3.2.4. Subjective Norm

Subjective norm scores reflect perceptions about normative behaviour within the relevant social group. Their social group (or salient referents) was described as ‘*people who are important to me/whose opinions I value'*, ‘*people like me*' and ‘*work colleagues'*. Four items reflected descriptive norms, i.e., the prevalence of help-seeking amongst important others (e.g., ‘*Most of the people who are important to me would ask for professional help if they were experiencing stress'*). Four further items measured injunctive norms, i.e., perceptions of what important others would approve/not approve of regarding help-seeking for stress (e.g., ‘*Most of my work colleagues would approve of me seeking professional help for stress if I needed it*'). The items were scored on a five-point scale: 1 (*completely false*) to 5 (*completely true*). A total score was computed with higher scores representing stronger beliefs about the social pressure to help-seek. Internal consistency was high (Cronbach's *α* = 0.81, McDonald's *ω* = 0.81).

#### 3.2.5. Perceived Behavioural Control

Four items (measured on a five-point scale) were used to measure respondents' perceived control over help-seeking for stress. The first two items measured respondents' self-efficacy in help-seeking; ‘*If I wanted to, I could seek professional help for stress',* 1 (*definitely false*) to 5 (*definitely true*), and ‘*For me seeking help from professionals when I am experiencing stress would be*'*…*1 (*possible*) to 5 (*impossible*).

Two further items measured respondents' controllability over help-seeking: ‘*If you were under stress, how much control do you believe you have over seeking professional help for this?'*, 1 (*no control*) to 5 (*complete control*), and *‘It is mostly up to me whether or not I decide to get help for stress',* 1 (*definitely false*) to 5 (*definitely true*). A computed score was calculated after reverse scoring *‘For me seeking help from professionals when I am experiencing stress would be' …*1 (*possible*) to 5 (*impossible*). Higher scores indicated greater perceived behavioural control. Internal consistency was acceptable (Cronbach's *α* = 0.75, McDonald's *ω* = 0.76).

#### 3.2.6. Behavioural Intention

Intention to seek help for stress was indexed using two items: ‘*If I experience stress, I will try to get help from a professional*,' 1 (*extremely unlikely*) to 5 (*extremely likely*), and ‘*I plan to get help from a professional if I ever experience stress*,' 1 (*definitely false*) to 5 (*definitely true*) (Cronbach's alpha = 0.85).

#### 3.2.7. Help-Seeking Behaviour

Help-seeking behaviour for stress was measured using two items: ‘Please estimate how often you sought professional help for stress over the past year' and ‘Please estimate how often you sought help for stress over the past year'. Participants responded to both items on a five-point Likert scale (*1 = never*, *2 = hardly ever*, *3 = sometimes*, *4 = frequently*, *5 = every day*). The two items were combined to create a composite help-seeking score (Cronbach's *α* = 0.71), with higher scores indicating more frequent help-seeking behaviour.

#### 3.2.8. Self-Reported 12-Month Stress

Self-reported stress over the past year was measured with a single item: ‘Please estimate how often you have been stressed over the past year'. Participants responded using a five-point Likert scale (*0 = never*, *1 = hardly ever*, *2 = sometimes*, *3 = frequently*, *4 = every day*). Higher scores indicated greater perceived stress.

### 3.3. Analytic Plan

To examine the relationships between attitudes, subjective norms, perceived behavioural control and help-seeking behaviour, we followed these steps: First, we estimated the direct effects (c paths) of these predictors on help-seeking behaviour. Next, we introduced the intermediary variable (behavioural intention) and estimated the direct effects (*a* paths) of the predictors on the intermediary, as well as the effects (*b* paths) of the intermediary on help-seeking behaviour, controlling for the intermediary (*c*′ paths). To measure indirect effects, we calculated the standardized indirect effects (*a* × *b*) from attitudes, subjective norms and perceived behavioural control to help-seeking behaviour through behavioural intention. Both help-seeking intention and 12-month stress were mean-centred before calculating their interaction term (*M* × *Z*) to assess its effect on help-seeking behaviour. Interaction effects were examined by probing the relationship between help-seeking intention and behaviour at low (−1 SD), average (mean) and high (+1 SD) levels of stress to understand conditional effects [36]. The analysis was conducted using Mplus 8.10 [37], with the maximum likelihood estimator and 10,000 bootstrapped samples to address nonnormality providing robust standard errors and confidence intervals.

## 4. Results

From the initial sample of 657 nurse participants, 108 cases contained missing data (coded as −99) and were treated using full information maximum likelihood estimation. Little's MCAR test indicated that the data were missing completely at random. Consequently, the analysis was performed on a final sample of 549 cases, representing 83.5% of the initial sample. The majority sample was white British (84.2%) and female (87.6%). Age was assessed across six age bands from under 20 to over 60. The modal age group was that from 50–59 (39%), then 40–49 (37.4%), followed by 30–39 (15.6%), the over 60 age group (3.8%), followed by 20–29 (4.2%). There were no endorsements for those under 20 years of age. Descriptive statistics indicated that most variables were approximately normally distributed, with skewness values within acceptable ranges (<±2) (see Table 2).

### 4.1. Correlations

Prior to model testing, Pearson correlations were examined among key TPB variables. Intention was significantly correlated with attitudes (*r* = 0.58, *p* < 0.001), subjective norms (*r* = 0.52, *p* < 0.001) and perceived behavioural control (*r* = 0.37, *p* < 0.001). In contrast, 12-month stress was not significantly correlated with any of the TPB variables (all *p* > 0.05).

### 4.2. Direct Effects and Moderation Analysis

The observed results showed statistically significant and positive relationships between attitudes (*β* = 0.41, SE = 0.04, 95% CI = 0.32–0.48), subjective norms (*β* = 0.30, SE = 0.05, 95% CI = 0.21–0.39) and behavioural intention. Perceived behavioural control was not significantly related to intention (*β* = 0.06, SE = 0.04, 95% CI = −0.02–0.14). There were statistically significant and positive relationships between 12-month stress (*β* = 0.33, SE = 0.04, 95% CI = 0.26–0.40) and behavioural intention (*β* = 0.48, SE = 0.05, 95% CI = 0.38–0.56) on help-seeking.

The results showed that the frequency of recent self-reported stress positively and significantly interacted with the relationship between help-seeking intention and recent help-seeking behaviour (*β* = 0.18, SE = 0.04, 95% CI = 0.11–0.24). The strength of the relationship between current intentions and recent help-seeking behaviour was found to increase along with the frequency of recent stress. Results are presented in Table 3.

### 4.3. Indirect Effects Analysis

The observed results showed that help-seeking intention served as an intermediary in the relationships between attitudes and help-seeking behaviour (*β* = 0.20, SE = 0.03, 95% CI = 0.14–0.25) and subjective norms and help-seeking behaviour (*β* = 0.15, SE = 0.03, 95% CI = 0.10–0.20), but not perceived behavioural control (*β* = 0.03, SE = 0.02, 95% CI = −0.01–0.07). Significance of the indirect effects was indicated when confidence intervals did not include zero. Therefore, attitudes and subjective norms (but not perceived behavioural control) are associated with higher help-seeking intention, which, in turn, predicts help-seeking behaviour over the previous 12 months (see Table 4).

### 4.4. Moderated Mediation Analysis

We examined whether the indirect effects of attitudes and subjective norms on help-seeking behaviour were moderated by the level of 12-month stress. Using the index of moderated mediation [38], we found that the 95% confidence intervals for the index of attitudes (*β* = 0.02, SE = 0.00, 95% CI = 0.09–0.23) and subjective norms (*β* = 0.02, SE = 0.01, 95% CI = 0.01–0.04) did not contain zero, indicating that the indirect effect of these variables on help-seeking behaviour is indeed moderated by 12-month stress. In contrast, the confidence interval for the index of perceived behavioural control included zero (*β* = 0.01, SE = 0.01, 95% CI = −0.00–0.02), indicating no significant effect. The conditional indirect effects of attitudes, subjective norms and perceived behavioural on help-seeking behaviour through behavioural intention at −1SD below, at the mean, and +1SD above the mean of 12-month stress. For attitudes and social norms, we found that the indirect effects were significant across all levels of stress, indicating a strengthening of the help-seeking pathway as stress increases. Perceived behavioural control was not significant at any level of stress. Full results are shown in Table 5.

## 5. Discussion

The findings of this study offer important insights into the psychosocial determinants that influence help-seeking behaviours among nurses when faced with stress. Positive attitudes and subjective norms, including perceptions of approval or disapproval from colleagues, are key predictors of help-seeking intentions. Participants who hold negative attitudes towards help-seeking and perceive lower levels of social support from colleagues are less likely to report intentions to seek help for stress. By contrast, perceived behavioural control, which refers to the individual's perceived ability to seek help, did not significantly predict help-seeking intentions. Our findings indicate that positive attitudes and subjective norms have a significant indirect effect on recent help-seeking behaviour through increased help-seeking intentions. Moreover, this relationship is moderated by the frequency of recent stress experiences, such that the association between positive attitudes/subjective norms and help-seeking behaviour is stronger among individuals who have recently experienced higher levels of stress.

Our findings contrast with a large volume of previous research which has applied the theory of planned behaviour to help-seeking. Such studies have tended to identify attitudes and perceived behavioural control as the most important factors for predicting intentions to seek help: for psychological difficulties in the general population [30, 39], in college students [40] and in patients with physical conditions [41]. In our study, perceived behavioural control did not emerge as significant, with subjective norms accompanying attitudes as the key predictors. The findings of Tomczyk et al. [42] may help explain this observation, as they highlight the nuanced nature of perceived behavioural control by distinguishing between its two facets: self-efficacy (confidence in one's ability to seek help) and controllability (perceived control over external circumstances). Their study demonstrated that while self-efficacy was a significant positive predictor of help-seeking intentions, controllability was not. This suggests that help-seeking behaviour may be influenced more by individuals' internal confidence in their ability to act than by their perceived situational control. Moreover, combined, they highlight that help-seeking intentions may be more closely tied to personal attitudes towards seeking support and perceived normative approval than perceptions of external barriers.

As such, our findings corroborate earlier evidence that both personal and social stigma are closely linked to organisational norms and prevent many health professionals from intending to or actually seeking help for psychological problems [6, 18, 31, 43]. Previous studies have shown that stigma discourages health professionals from seeking help or admitting to help-seeking. Our study is the first to apply a model of health behaviour to understand the key psychological drivers affecting stress help-seeking in nurses whilst also accounting for the frequency of stress.

Workplace anti-stigma interventions targeting people's knowledge and attitudes to mental health can be effective [44]. However, interventions are more impactful when addressing systemic organisational causes as well [45]. Our findings are consistent with this; they suggest that interventions to combat the stigma of stress help-seeking in nurses must not only target personal attitudes but also challenge the dominant cultural norms within organisations that act as barriers to help-seeking [46]. However, a recent systematic meta-review by McCullock and Scrivano [47] highlights a persistent gap in the literature, noting a lack of research on multilevel approaches. They emphasise the need to prioritise multicomponent strategies that address mental illness stigma across individual, organisational and societal levels. For this to happen, there needs to be a genuine commitment from healthcare managers to establish and maintain a balance between organisational production goals and the psychological health of staff. Dollard and McTernan [48] refer to this balance as a psychosocial safety climate. A strong organisational psychosocial safety climate should also include robust communication and participation between management and staff regarding psychological health.

### 5.1. Limitations

This study's cross-sectional design relied on retrospective self-reports of help-seeking behaviour for stress over the previous 12 months, which may compromise measurement accuracy due to potential recall bias. While help-seeking is a sensitive and infrequent behaviour that may support recall accuracy, retrospective self-reporting, particularly when relying on a single-item stress measure, may reduce reliability and introduce bias. Additionally, the self-report nature of the data means that accuracy depends on participants' honesty and recall ability. Self-reported data are inherently less objective than behavioural measures, such as recorded visits to occupational health. However, help-seeking is a sensitive and personal activity, and collecting objective data on confidential visits for occupational stress would have been challenging, especially in work environments where stigma around help-seeking may exist.

Future studies could enhance the validity of self-reported help-seeking measures in several ways. Adopting a longitudinal design would provide stronger evidence of temporal relationships among variables and strengthen causal interpretation. The validity of self-reported help-seeking could also be improved by asking respondents to specify the types of help-seeking they engaged in, as this behaviour can take various forms.

It should be noted that some variables in this study were measured with a limited number of items, including perceived behavioural control, which showed a nonsignificant effect. This may reflect measurement limitations and warrants further exploration. However, many large-scale, community-based studies and high-quality epidemiological surveys, such as the National Epidemiologic Survey on Alcohol and Related Conditions (NESARC) in the United States and the Adult Psychiatric Morbidity Survey (APMS) in the United Kingdom, successfully use single-item or two-item measures to assess various mental health outcomes and behaviours [49, 50]. These measures can provide reliable and valid data, particularly in large-scale surveys where brevity is essential to maintain high response rates and effectively inform policymakers. Nonetheless, future research could benefit from using more comprehensive measures to better capture nuanced experiences and complexities.

The study sample was also a small proportion of the NMC membership (approximately 716,000 members). Thus, the data reported here are from a self-selecting group whose attitudes and experiences may differ from those of the broader nursing and midwifery professions in the United Kingdom. The predominantly white and female sample further limits generalisability. Evidence suggests that people from non-white ethnicities in the United Kingdom may be less inclined to use formal mental health support [51, 52]; hence, the current findings may reflect a Caucasian-biased outcome, limiting generalisability to more diverse populations. Future studies should aim to recruit more ethnically and gender-diverse participants to enhance relevance.

## 6. Conclusions

Nurses are more likely to intend to seek help for stress when they have a positive attitude towards help-seeking and believe that those around them would approve of and engage in help-seeking themselves. By contrast, perceived behavioural control was not a significant predictor of these intentions. Current help-seeking intentions were also stronger among those who had sought help in the previous 12 months. Furthermore, the relationship between attitudes, social norms, help-seeking intentions and help-seeking behaviour is stronger in individuals who have experienced frequent stress recently. These findings suggest that interventions aimed at reducing the stigma associated with help-seeking for stress within healthcare organizations should target both the personal attitudes of nurses and allied health professionals and the cultural norms within these institutions. Additionally, universities should consider including stress management techniques and demystifying help-seeking as part of the healthcare curriculum, equipping future professionals with both the skills and mindset to manage stress effectively, fostering a culture of openness and support in their workplaces.

## Figures and Tables

**Table 1:** Demographic variables of sample.

Variable	*N*	%	Missing
Gender			13
Female	481	87.6	
Male	55	10.0	
Band			113
< 5	3	0.5	
5	134	24.4	
6	131	23.9	
7	126	23.0	
8	33	6.0	
Senior management	3	1.6	
Ethnicity			4
White British	462	84.2	
White other	47	8.6	
Black/Black mixed	24	4.4	
Asian/Asian mixed	12	2.2	

**Table 2:** Descriptive statistics for main variables of interest and demographics.

Items	Mean	SD	Min-Max	Skew
Attitudes	38.01	11.70	7–63	−0.184^∗∗^
Subjective norms	24.32	5.51	8–40	−0.181^∗∗^
Perceived behavioural control	16.04	3.01	4–20	−0.748^∗∗^
Behavioural intention	5.61	2.20	2–10	0.188^∗∗^
12-month stress	3.57	0.69	1–5	−0.242^∗∗^
Help-seeking behaviour	3.45	1.80	2–8	1.03^∗∗^

*Note:* Estimates of skew were statistically significant at *p* < 0.01.

^∗∗^ *p* < 0.001.

**Table 3:** Standardized direct and interaction effects on behavioural intention and help-seeking.

	Behavioural intention	Help-seeking
	β (S.E) 95% CI	β (S.E) 95% CI
Attitudes	**0.41 (0.04) 0.32–0.48**	0.07 (0.04) −0.02–0.16
Subjective norms	**0.30 (0.05) 0.21–0.39**	−0.02 (0.05) −0.11–0.07
Perceived behavioural control	0.06 (0.04) −0.02–0.14	0.06 (0.04) −0.12–0.15
12-month stress^a^	—	**0.33 (0.04) 0.26–0.40**
Behavioural intention^b^	—	**0.48 (0.05) 0.38–0.56**
Interaction (^a^ × ^b^)	—	**0.18 (0.04) 0.11–0.24**
R^2	**0.41,** **p** < 0.001	**0.40,** **p** < 0.001

*Note: β* = standardized beta coefficient. The bold values denote that the relationships/statistics are significant.

Abbreviations: 95% CI = 95% confidence intervals, S.E = standard error.

^a^12-month stress.

^b^Behavioural intention.

**Table 4:** Indirect effects of attitudes, subjective norms and perceived behavioural control on help-seeking via behavioural intention.

From item	Via	*β* (S.E) 95% CI	To item
Attitudes	Behavioural intention	**0.20 (0.03) 0.14–0.25**	Help-seeking
Subjective norms	Behavioural intention	**0.15 (0.03) 0.10–0.20**	Help-seeking
Perceived control	Behavioural intention	0.03 (0.02) −0.01–0.07	Help-seeking

*Note: β* = standardised beta coefficient. The bold values denote that the relationships/statistics are significant.

Abbreviations: 95% CI = 95% confidence intervals, S.E = standard error.

**Table 5:** Index of moderated mediation and conditional indirect effects at different levels of 12-month stress.

From item	(βeta)	(SE)	95% CI
Attitudes (IMM)	**0.02**	**0.00**	**0.09–0.23**
Low stress	**0.08**	**0.01**	**0.05–0.10**
Mean stress	**0.09**	**0.01**	**0.06–0.12**
High stress	**0.10**	**0.01**	**0.07–0.13**
Subjective norms (IMM)	**0.02**	**0.01**	**0.01–0.04**
Low stress	**0.12**	**0.02**	**0.10–0.16**
Mean stress	**0.13**	**0.02**	**0.11–0.19**
High stress	**0.15**	**0.03**	**0.12–0.22**
Perceived behavioural control (IMM)	0.01	0.01	−0.00–0.02
Low stress	0.04	0.03	−0.01–0.09
Mean stress	0.05	0.03	−0.10–0.12
High stress	0.06	0.04	−0.01–0.14

*Note: β* = standardised beta coefficient. The index of moderated mediation (IMM) quantifies the extent to which the strength of the indirect effects varies with the moderator (stress levels). The bold in the tables denote that the relationships/statistics are significant.

Abbreviations: 95% CI = 95% confidence intervals, S.E = standard error.

## Data Availability

The raw data supporting the findings of this study are available from the corresponding author upon reasonable request.
